# A novel approach for designing hands‐on training programs using Quick Reference code‐linked videos

**DOI:** 10.1002/jdd.13448

**Published:** 2024-01-07

**Authors:** Jesse W. Manton, Ned L. Nix, Fatima Mashkoor, David K. Lam

**Affiliations:** ^1^ Department of Oral and Maxillofacial Surgery University of the Pacific Arthur A. Dugoni School of Dentistry San Francisco California USA

**Keywords:** curriculum innovation, educational methodology, educational technology, teaching methods

## Abstract

Providing training on the proper use of technology in the context of a large number of learners and limited faculty availability is a constant challenge to dental schools. We found the implementation of a QR‐code‐based autonomous program design to be efficient and effective at providing trainees hands‐on training with newly installed perioperative equipment.

## 1 PROBLEM

Technology is evolving at a blistering pace and educational programs continue to explore how best to incorporate these advances.^1^ Leveraging technology to help facilitate training programs for large numbers of learners could be of significant value in the context of limited faculty availability. Our department recently installed new surgical and perioperative equipment and was challenged to develop training resources for all of our rotating trainees in order to prepare them for the safe delivery of patient care using this equipment.

## 2 SOLUTION

**FIGURE 1:**
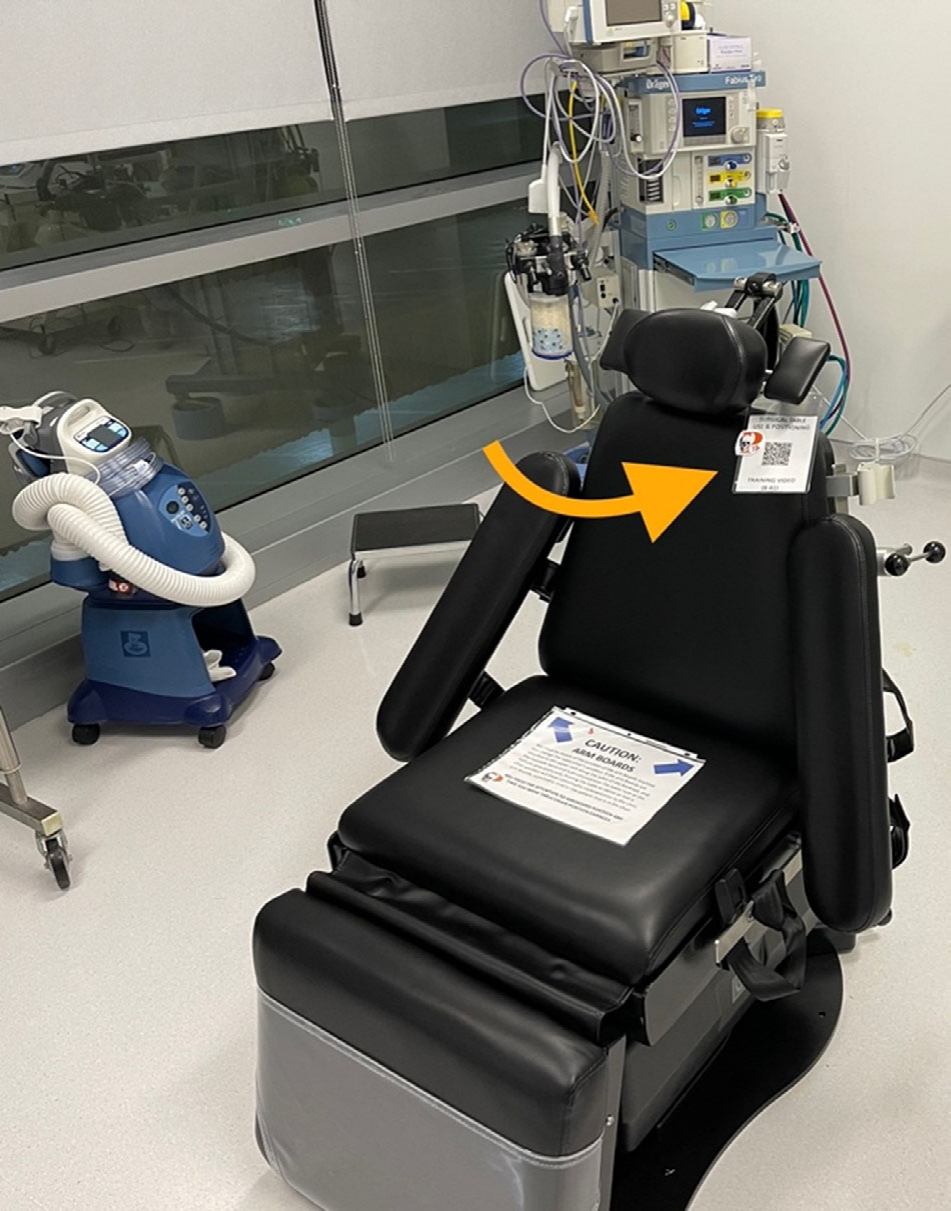
Learning station on protocols for use of a new surgical table, displaying Quick Response (QR) code labels (orange arrow).

We developed a novel hands‐on training program for newly installed equipment using Quick Reference (QR) code technology.^2^ Equipment included surgical tables, surgical motors, patient monitors, nitrous oxide sedation units, and general anesthesia machines, which were identified as having a risk for equipment malfunction and patient harm if used or damaged by untrained providers. Short 5‐min training videos covering protocols for proper setup, use, disinfection, and storage of equipment were produced using footage captured with an Apple iPhone 12 Pro and iMovie. Videos were uploaded to the Mediasite server and additional training videos were sourced from YouTube. Shareable URL links for these videos were input into a free QR code‐generating website.^3^ Small labels were designed for each piece of equipment using Microsoft Word to display these QR codes. Labels were placed in clear badge holders and affixed to the equipment (Figure 1).

As part of implementing this innovation into our clinic, we developed a training program for 148 first‐year dental students during their existing preclinical block rotations using 24 one‐hr sessions for groups of approximately six students at a time across eight consecutive weeks. A faculty member spent 10 min setting up and breaking down the learning stations each week. Upon arrival at the clinic, students signed in and scanned the first QR code with their personal device's camera application, linking them to an introductory video that initiated their self‐directed session and eventually led them to the next station. Training videos directed students to interact with equipment at each station, (e.g., change table positioning and assemble nitrous oxide unit) emphasizing hands‐on kinesthetic learning.^4^, ^5^ At the final station, students were directed to complete a survey before exiting the clinic. In response to feedback, a 30‐min drop‐in Question and Answer (Q&A) session was made available for students each week. Student learning was tested using multiple‐choice questions on the block's final exam.

## 3 RESULTS

**FIGURE 2:**
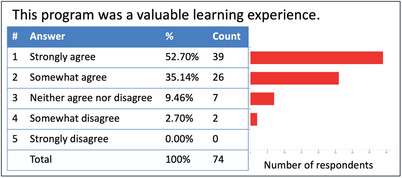
Survey results regarding the perceived value of the learning experience. (50% Response Rate, 74/148).

**FIGURE 3:**
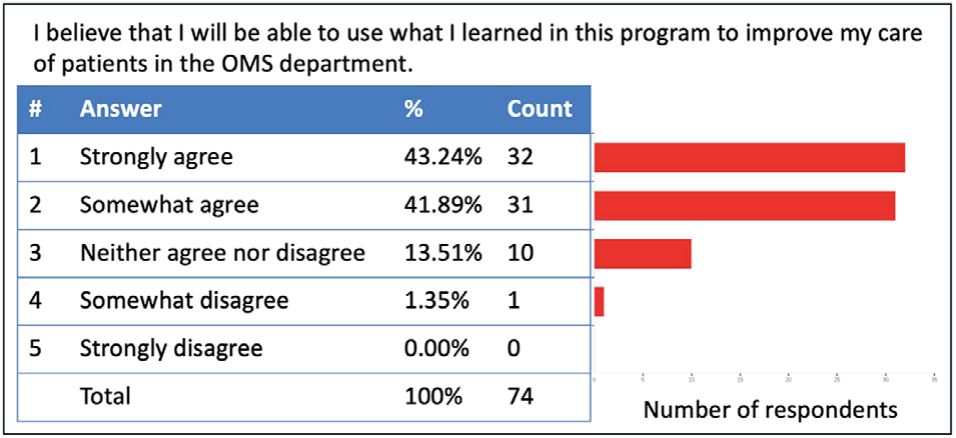
Survey results regarding the translation of student learning toward clinical application. (50% Response Rate, 74/148).

This autonomous program design required only 1.5 hr of faculty time rather than the 25 hr that would have been required for eight weeks of instructor‐facilitated sessions. Survey results found that 87% of students agreed that sessions were valuable learning experiences (Figure 2) and 85% agreed that they could use what they learned to improve patient care (Figure 3). Survey results indicated that 86% of students enjoyed the rotation. The optional Q&A sessions were not attended by any students. We found this novel approach to providing hands‐on training to be effective for teaching and efficient with faculty time. This innovation also created a continuously available point‐of‐care training resource for all rotating providers.
